# Comparison of monoclonal antibodies targeting CD38, SLAMF7 and PD-1/PD-L1 in combination with Bortezomib/Immunomodulators plus dexamethasone/prednisone for the treatment of multiple myeloma: an indirect-comparison Meta-analysis of randomised controlled trials

**DOI:** 10.1186/s12885-021-08588-9

**Published:** 2021-09-06

**Authors:** Wu Ye, Xia Wu, Xiaoyan Liu, Xue Zheng, Jili Deng, Yuping Gong

**Affiliations:** Department of Hematology, West China Hospital, Sichuan University, Chengdu, No.37 GuoXue Xiang, Chengdu, 610041 Sichuan Province China

**Keywords:** Monoclonal antibodies, CD38, SLAMF7, PD-1/PD-L1, Meta-analysis

## Abstract

**Background:**

Many clinical trials have assessed the effect and safety of monoclonal antibodies (MAbs) in combination with proteasome inhibitors or immunomodulators plus dexamethasone/prednisone for the treatment of multiple myeloma (MM). The treatment outcomes of comparing different MAbs in combination with the above-mentioned agents remained unclear. We performed the meta-analysis to indirectly compare the effect and safety of MAbs targeting CD38, SLAMF7, and PD-1/PD-L1 in combination with bortezomib/immunomodulators plus dexamethasone/prednisone for patients with MM.

**Methods:**

We searched thoroughly in the databases for randomised controlled trials (RCTs) in which at least one of the three MAbs were included. We included eleven eligible RCTs with 5367 patients in the meta-analysis. Statistical analysis was carried out using StataMP14 and Indirect Treatment Comparisons software.

**Results:**

We calculated hazard ratios (HRs) for overall survival (OS) and progression-free survival (PFS) and relative risk (RR) for overall response rate, complete response (CR) or better, very good partial response (VGPR) or better, VGPR, partial response, stable disease, and grade 3 or higher adverse events among the three groups. The HRs for PFS of the CD38 group vs SLAMF7 group, CD38 group vs PD-1/PD-L1 group, and SLAMF7 group vs PD-1/PD-L1 group were 0.662 (95%CI 0.543–0.806), 0.317 (95%CI 0.221–0.454), and 0.479 (95%CI 0.328–0.699), respectively. The HR for OS of the CD38 group vs SLAMF7 group was 0.812 (95%CI 0.584–1.127). The RR for CR or better in the CD38 group vs SLAMF7 group was 2.253 (95%CI 1.284–3.955). The RR for neutropenia of the CD38 group vs SLAMF7 group was 1.818 (95%CI 1.41–2.344).

**Conclusions:**

Treatment with the CD38 group had longer PFS and better treatment response than that with the SLAMF7 or PD-1/PD-L1 group. In addition, the SLAMF7 group prolonged PFS compared with the PD-1/PD-L1 group and was associated with a lower incidence of grade 3 or higher neutropenia than the CD38 and PD-1/PD-L1 group. In conclusion, MAbs targeting CD38 are the best, followed by those targeting SLAMF7; MAbs targeting PD-1/PD-L1 are the worst when in combination with bortezomib/immunomodulators plus dexamethasone/prednisone for the treatment of MM.

**Supplementary Information:**

The online version contains supplementary material available at 10.1186/s12885-021-08588-9.

## Background

Multiple Myeloma (MM) is a malignant plasma cell disease, which is the second most common malignancy in the hematological system. It accounted for 1% of all reported tumours and 13% of hematological cancers. There were approximately 9000 newly diagnosed cases per year in Europe [1]. The cluster of differentiation 38 (CD38) protein is a enzyme with multiple functions including degrading nicotinamide adenine dinucleotide (NAD) and regulating cellular NAD homeostasis; it has been discovered that CD38 was a cell surface marker of hematological malignancies such as MM [2]. CD38 was expressed at high levels in MM cells but at low levels in normal blood cells. Monoclonal antibodies (MAbs) targeting CD38 such as daratumumab approved by the FDA have been applied in the treatment of MM [3]. SLAMF7, also known as CD319, CS1, and CRACC, was high-expressed in MM cells and regarded as a target for specific anti-tumour immune responses [4–6]. The MAbs targeting the surface SLAMF7 glycoprotein of MM cells such as elotuzumab improved the prognosis of MM patients when administered in combination with proteasome inhibitors or immunomodulators [7, 8]. Apart from CD38 and SLAMF7, the programmed cell death protein 1 (PD-1) and its ligand - programmed cell death ligand 1 (PD-L1) also played a significant role in MM cells. The pathway of PD-1/PD-L1 was a significant negative modulator of immune responses, which was over up-regulated in MM cells and was one of the critical factors for immune escape of tumour cells [9]. In the treatment of MM patients, the MAbs targeting PD-1/PD-L1, including pembrolizumab, durvalumab and nivolumab, had been reported [10]; unfortunately, anti-PD-1/PD-L1 antibodies had poor therapeutic effect as a single agent [11].

Many clinical trials have assessed the effect and safety of using MAbs in combination with proteasome inhibitors or immunomodulators plus dexamethasone/prednisone in the treatment of MM. However, there has been no clinical study comparing the effects of different MAbs directed to different targets in combination with the above-mentioned drugs. Thus, we performed a meta-analysis to indirectly compare the effect and safety of the MAbs targeting CD38, SLAMF7, and PD-1/PD-L1 in combination with bortezomib/immunomodulators plus dexamethasone/prednisone in patients with MM.

## Methods

We performed this meta-analysis based on PRISMA statements, and the protocol was registered with PROSPERO, number CRD42020171456.

### Study inclusion and exclusion criteria

Inclusion criteria: 1. The three experimental groups were the MAbs targeting CD38, SLAMF7, or PD-1/PD-L1 in combination with bortezomib/immunomodulators plus dexamethasone/prednisone; the control groups were blank, placebo, or conventional agents. 2. Participants were patients diagnosed with MM. 3. Study outcomes included survival outcomes, treatment responses, and common adverse events. 4. Studies must be randomised controlled trials (RCTs).

Exclusion criteria: 1. The same study was published repeatedly. 2. The studies had incomplete results and we obtained no supplementary data after contacting the author. 3. The studies involved basic research and animal experimental research. 4. Studies had an insufficient follow-up time or more than 20% of the patients included in the study were lost to follow-up.

### Search strategy and screening

Systematic studies searches were performed using PubMed, Embase, Medline, Web of Science, Cochrane Library and Chinese Biomedical Database (before 26 Feb 2020). The retrieval terms and methods were as following: 1#: “Monoclonal Antibodies” OR “CD38” OR “PD-1/PD-L1” OR “SLAMF7” OR “CD319” OR “CS1” OR “19A” OR “CRACC” OR “Daratumumab” OR “Isatuximab” OR “MOR202” OR “TAK-079” OR “Pembrolizumab” OR “Nivolumab” OR “Elotuzumab”, 2#: “Bortezomib” OR” Immunomodulatory” OR “Lenalidomide” OR “Pomalidomide” OR “Thalidomide”, 3#:“Dexamethasone” OR “Prednisone”, 4#: “Multiple Myeloma”, 5#: 1#AND2#AND3#AND4#. Two authors independently reviewed the titles and abstracts to screen potentially eligible studies; subsequently, two authors reviewed the full text to screen qualified articles independently. Disagreements between authors were solved by consensus or consultation with a third author.

### Critical appraisal of the included studies

According to ‘Cochrane collaboration’s tool for assessing the risk of bias’, the evaluation contents include the following aspects: 1. Blind methods; 2. Randomized methods; 3. Allocation concealment; 4. Incomplete outcomes; 5. Selective reporting; 6. Other biases. Two authors assessed the quality of the studies independently.

### Data extraction

All data from the included studies were extracted independently by two authors. We extracted the information including the number of patients, the experimental and control groups, follow-up time, survival outcomes, treatment responses of patients, and common hematological and non-hematological adverse events.

In the meta-analysis, progression-free survival (PFS) was the primary endpoint [12]; the secondary endpoints included overall survival (OS), overall response rate (ORR), complete response (CR) or better, very good partial response (VGPR) or better, VGPR, partial response (PR), stable disease (SD), and grade 3 or higher common hematological and non-hematological adverse events.

### Statistical analysis

To estimate the pooled hazard ratios (HRs) for survival outcomes or relative risk (RR) for the treatment response and the incidence of adverse events, we conducted a conventional meta-analysis by using the StataMP14 software. Statistical heterogeneity was assessed by I^2^ statistic. If I^2^ was more than 50%, we considered that there was distinct heterogeneity among studies. We selected the random-effects model to estimate the effect values when distinct heterogeneity existed, otherwise the fixed-effects model was selected [13]. HRs with their corresponding 95% confidence intervals (CI) for OS and PFS were utilized to compare the prognostic survival; RR with 95% CI was utilized to compare the treatment responses of patients and the incidence of adverse events [12].

Finally, we used the Indirect Treatment Comparisons (ITC) software to compare HR or RR generated as described above.

## Results

### Screening results and characteristics of the included studies

**Fig. 1:**
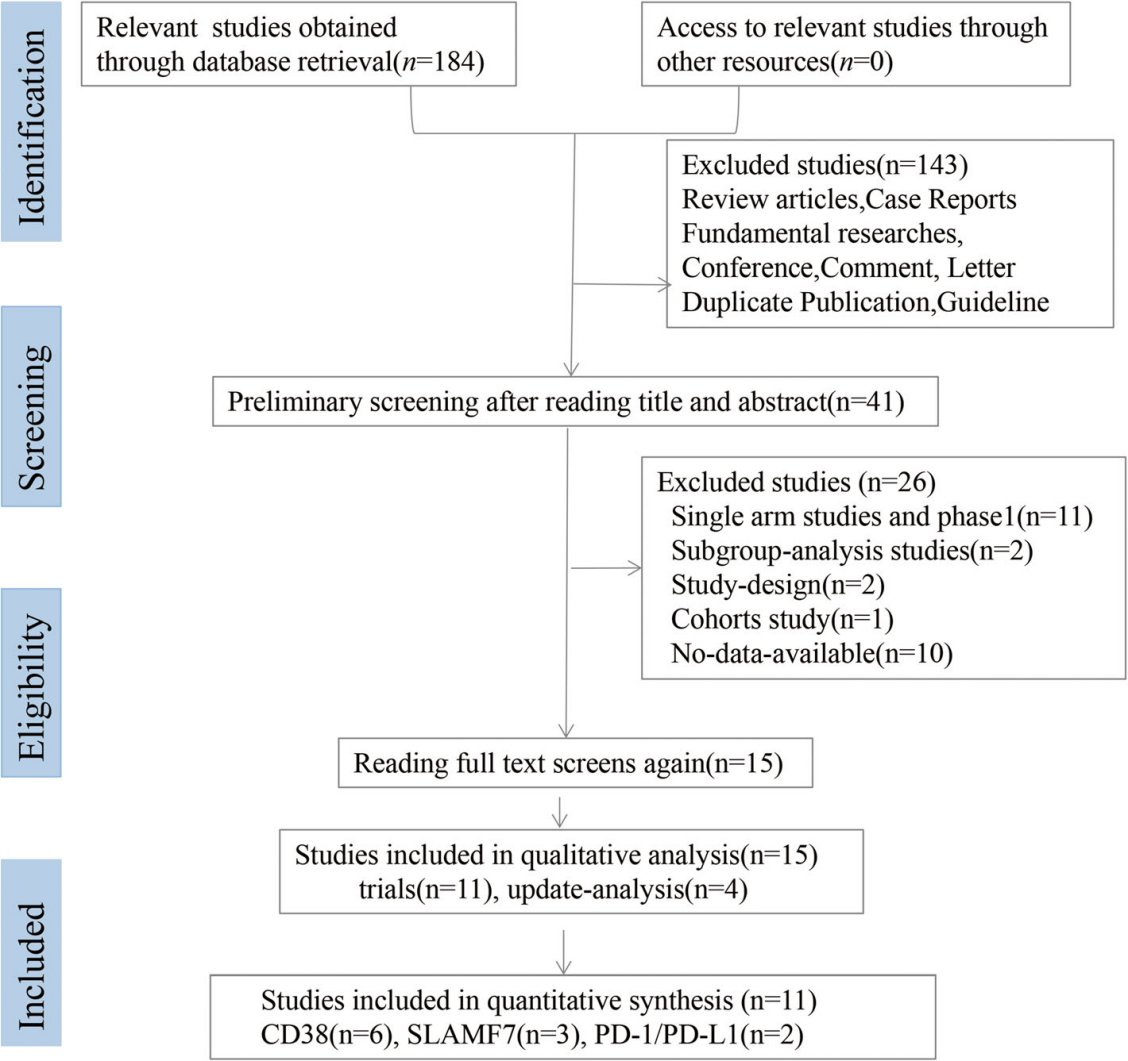
The screening process of randomised controlled trials included in the meta-analysis

We retrieved a total of 184 articles from PubMed, Embase, Medline, Web of Science, Cochrane Library and Chinese Biomedical Database. The process of study selection was shown in Fig. 1. Preliminary screening after reading titles and abstracts, excluding review articles, basic research articles, case reports, conferences, comments, letters, guidelines, and duplicate publications, resulted in the selection of 41 studies. After reading the full-text of studies again and following the exclusion of single-arm and phase 1, subgroup-analysis, study-design, cohorts, and no-data-available studies, we obtained 15 articles totally that included 11 RCTs and 4 update-analysis studies [14–17]. Finally, 11 RCTs with 5367 patients were included in quantitative synthesis. Six RCTs [18–23] investigated the effect and safety of the MAbs targeting CD38 (including daratumumab, and isatuximab), three [24–26] investigated the effect and safety of the MAbs targeting SLAMF7 (including elotuzumab), and two [27, 28] investigated the effect and safety of the MAbs targeting PD-1/PD-L1 (including pembrolizumab). Characteristics of the included studies were presented in Table 1 and characteristics of the patients at baseline were presented in Additional file 1.

**Table 1:** Characteristics of the included studies

Study	Phase	Number of patient	Treatment regimens	Median follow-up Time(M)	Primary endpoint	Median PFS(M)	PFS rate	Median treatment Time(M)
Dimopoulos(2016) POLLUX Dimopoulos(2018) POLLUX	III	569	T:daratumumab16mg/kg + lenalidomide25mg + dexamethasone40mg C:lenalidomide25mg + dexamethasone40mg	T:13.5 C:13.5 T:25.4 C:25.4	PFS	T:N C:18.4 T:N C:17.5	12 Months T:83.2%C:60.1% 24 Months T:68.0%C:40.9%	T:24.5 C:16.0
Palumbo(2016) CASTOR Palumbo(2018) CASTOR	III	498	T:daratumumab 16 mg/kg +bortezomib1.3 mg/m^2^ + dexamethasone20mg C:bortezomib1.3 mg/m^2^ + dexamethasone20mg	T:7.4 C:7.4 T:19.4 C:19.4	PFS	T:N C:7.2 T:16.7 C:7.1	12 Months T:60.7%C:26.9% 18 Months T:48% C:7.9%	T:13.4 C:5.2
Facon(2019) NCT02252172	III	737	T:daratumumab16mg/kg + lenalidomide25mg + dexamethasone40mg C:lenalidomide25mg + dexamethasone40mg	T:28 C:28	PFS	T:N C:31.9	30 months T:N C:N	T:25.3 C:21.3
Mateos(2017) ALCYONE Maria(2019) ALCYONE	III	706	T:daratumumab16mg/kg + bortezomib1.3 mg/m^2^ + melphalan9mg/m^2^ + prednisone 60 mg/m^2^ C:bortezomib1.3 mg/m^2^ + melphalan9mg/m^2^ + prednisone 60 mg/m^2^	T:16.5 C:16.5 T:40.1 C:40.1	PFS	T:N C:18.1 T:36.4 C:19.3	18 months T:71.6%C:50.2% 36 months T:50.7%C:18.5%	T:14.7 C:12
Michel (2019) ICARIA-MM	III	307	T:isatuximab10mg/kg + pomalidomide4mg + dexamethasone 40 mg C:pomalidomide4mg + dexamethasone40mg	T:11.6 C:11.6	PFS	T:11.5 C:6.5	T:N C:N	T:9.6 C:5.6
Philippe (2019) CASSIOPEIA	III	1085	T:daratumumab16mg/kg + bortezomib1.3 mg/m^2^ + thalidomide100mg + dexamethasone40mg C:bortezomib1.3 mg/m^2^ + thalidomide100mg + dexamethasone40mg	T:18.8 C:18.8	SCR	T:N C:N	T:N C:N	T:8.9 C:8.7
Mateos(2019) KEYNOTE-183	III	249	T: pembrolizumab 200 mg +pomalidomide 4 mg + dexamethasone40mg C:pomalidomide 4 mg + dexamethasone40mg	T:8.1 C:8.1	PFS OS	T:5.6 C:8.4	6 months T:48% C:60%	T:4.1 C:4.2
Usmani(2019) KEYNOTE-185	III	301	T:pembrolizumab200mg + lenalidomide25mg + dexamethasone40mg C:+lenalidomide25mg + dexamethasone40mg	T:6.6 C:6.6	PFS	T:N C:N	6 months T:82% C:85%	T:4.4 C:N
Andrzej(2016) NCT01478048	II	152	T:Elotuzumab10mg/kg + bortezomib1.3 mg/m^2^ + dexamethasone20mg C:bortezomib1.3 mg/m^2^ + dexamethasone20mg	T:15.9 C:11.7	PFS	T:9.7 C:6.9	24 months T:18% C:11%	T:N C:N
Meletios(2018) ELOQUENT-3	II	117	T:Elotuzumab10-20 mg/kg + pomalidomide4mg + dexamethasone40mg C:pomalidomide4mg + dexamethasone40mg	T:9.1 C:9.1	PFS	T:10.3 C:4.7	T:N C:N	T:8.4 C:4.7
Sagar(2015) ELOQUENT-2 Dimopoulos(2018) ELOQUENT-2	III	646	T:Elotuzumab10mg/kg +lenalidomide25mg + dexamethasone40mg C:lenalidomide25mg + dexamethasone40mg	T:24.5 C:24.5 T:46 C:48	PFS ORR	T: 19.4 C:14.9 T:N C:N	12 months T:68%C:57% 24 months T:41%C:27% 48 months T:21%C:14%	T: 19 C:14

*Abbreviations*: *PFS* progression-free survival, *ORR* overall response rate, *SCR* stringent complete response, *N* not available/reached, *T* trail, *C* control, *M* month

According to ‘Cochrane collaboration’s tool for assessing the risk of bias’, we assessed the quality of the included studies, with the result indicating that the quality of all were high; the details of the quality assessment were presented in Table 2.

**Table 2:** Quality assessment of the included studies according to Cochrane collaboration’s tool for assessing risk of bias

Included studies	Randomized methods	Blind methods	Allocation concealment	Incomplete outcome data	Selective reporting	Other biases
POLLUX	Low risk	Unclear	Unclear	Low risk	Unclear	Unclear
CASTOR	Low risk	Unclear	Unclear	Low risk	Unclear	Unclear
NCT02252172	Low risk	Unclear	Unclear	Low risk	Unclear	Unclear
ALCYONE	Low risk	High risk	Unclear	Low risk	Unclear	Unclear
ICARIA - MM	Low risk	High risk	Unclear	Low risk	Unclear	Unclear
CASSIOPEIA	Low risk	High risk	Unclear	Low risk	Unclear	Unclear
KEYNOTE-183	Low risk	High risk	Unclear	High risk	Unclear	Unclear
KEYNOTE-185	Low risk	High risk	Unclear	High risk	Unclear	Unclear
NCT01478048	Low risk	High risk	Unclear	Low risk	Unclear	Unclear
ELOQUENT-3	Low risk	Unclear	Unclear	Low risk	Unclear	Unclear
ELOQUENT-2	Low risk	Unclear	Unclear	Low risk	Unclear	Unclear

### Progression-free survival

**Fig. 2:**
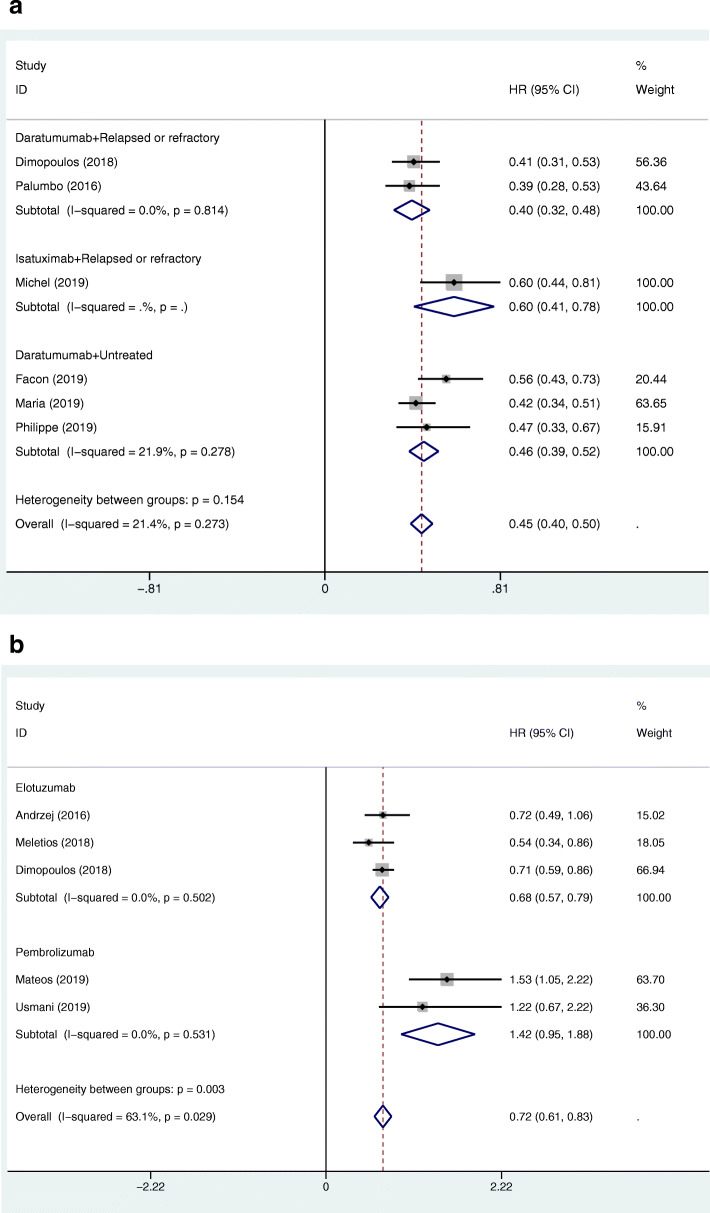
**a**. Forest plots of the pooled HRs for the PFS of the patients with relapsed or refractory MM or untreated MM in the MAbs targeting CD38 (including daratumumab, and isatuximab) group versus control group. The HR < 1 favours the MAb group. The size of the blocks or diamonds represents the weight of the fixed effects model in the meta-analysis. **b** Forest plots of the pooled HRs for the PFS of the patients with MM in the MAbs targeting SLAMF7 (including elotuzumab) and PD-1/PD-L1 (including pembrolizumab) groups versus their corresponding control groups. The HR < 1 favours the MAb group. The size of the blocks or diamonds represents the weight of the fixed effects model in the meta-analysis

All of the 11 RCTs provided PFS and HRs [12]. First, we synthesized the pooled HRs of the MAbs targeting CD38, SLAMF7, and PD-1/PD-L1 groups versus their corresponding control groups by StataMP14 software (Fig. 2a, b). The pooled HRs for the PFS of the MAbs targeting CD38, SLAMF7, and PD-1/PD-L1 groups vs their corresponding control groups were 0.45 (95%CI 0.40–0.50), 0.68 (95%CI 0.57–0.79), and 1.42 (95%CI 0.95–1.88), respectively. Second, we indirectly calculated the HRs for the PFS of the MAbs targeting CD38 group vs SLAMF7 group, CD38 group vs PD-1/PD-L1 group, and SLAMF7 group vs PD-1/PD-L1 group as 0.662 (95%CI 0.543–0.806), 0.317 (95%CI 0.221–0.454), and 0.479 (95%CI 0.328–0.699), respectively. The MAbs targeting CD38 group and SLAMF7 group prolonged PFS compared with their corresponding control groups, and the MAbs targeting CD38 group showed a longer PFS than the SLAMF7 group by indirect comparison. In contrast, the MAbs targeting PD-1/PD-L1 group performed the worst among the three groups. In a subgroup analysis of the MAbs targeting CD38 group, the HR for the PFS of the daratumumab group vs control group was 0.40 (95%CI 0.32–0.48) as compared to 0.60 (95%CI 0.41–0.78) of the isatuximab group vs control group, suggesting that the daratumumab group may result in longer PFS than the isatuximab group in patients with relapsed or refractory MM.

### Overall survival

**Fig. 3:**
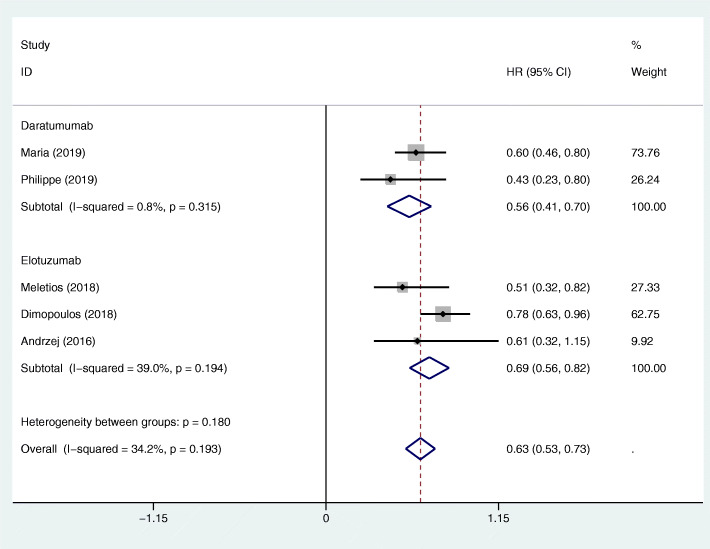
Forest plots of the pooled HRs for the OS of the patients with MM in the MAbs targeting CD38 (including daratumumab) and SLAMF7 (including elotuzumab) groups versus their corresponding control groups. The HR < 1 favours the MAb group. The size of the blocks or diamonds represents the weight of the fixed effects model in the meta-analysis

Two RCTs about the MAbs targeting CD38 and three RCTs about the MAbs targeting SLAMF7 provided OS and HR with the pooled HRs being 0.56 (95%CI 0.41–0.70) and 0.69 (95%CI 0.56–0.82), respectively (Fig. 3). The HR for the OS of the MAbs targeting CD38 group vs the MAbs targeting SLAMF7 group was 0.812 (95%CI 0.584–1.127) by indirect comparison. There was no significant difference in the OS between the two groups.

### ORR, CR or better, VGPR or better, VGPR, PR, SD

**Fig. 4:**
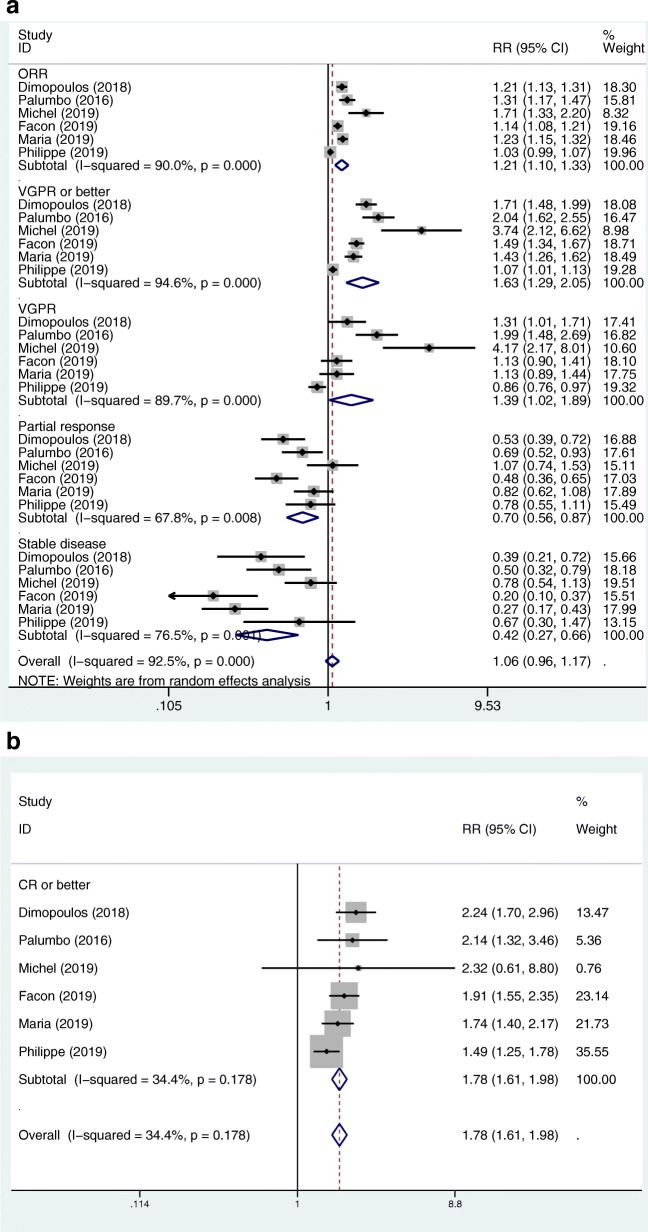
**a**. Forest plots of the pooled RRs for the ORR, VGPR or better, VGPR, partial response, and stable disease of the patients with MM in the MAbs targeting CD38 group versus control group. The RR > 1 favours the MAb group. The size of the blocks or diamonds represents the weight of the random effects model in the meta-analysis. **b** Forest plots of the pooled RR for the complete response or better of the patients with MM in the MAbs targeting CD38 group versus control group. The RR > 1 favours the MAb group. The size of the blocks or diamonds represents the weight of the fixed effects model in the meta-analysis

**Fig. 5:**
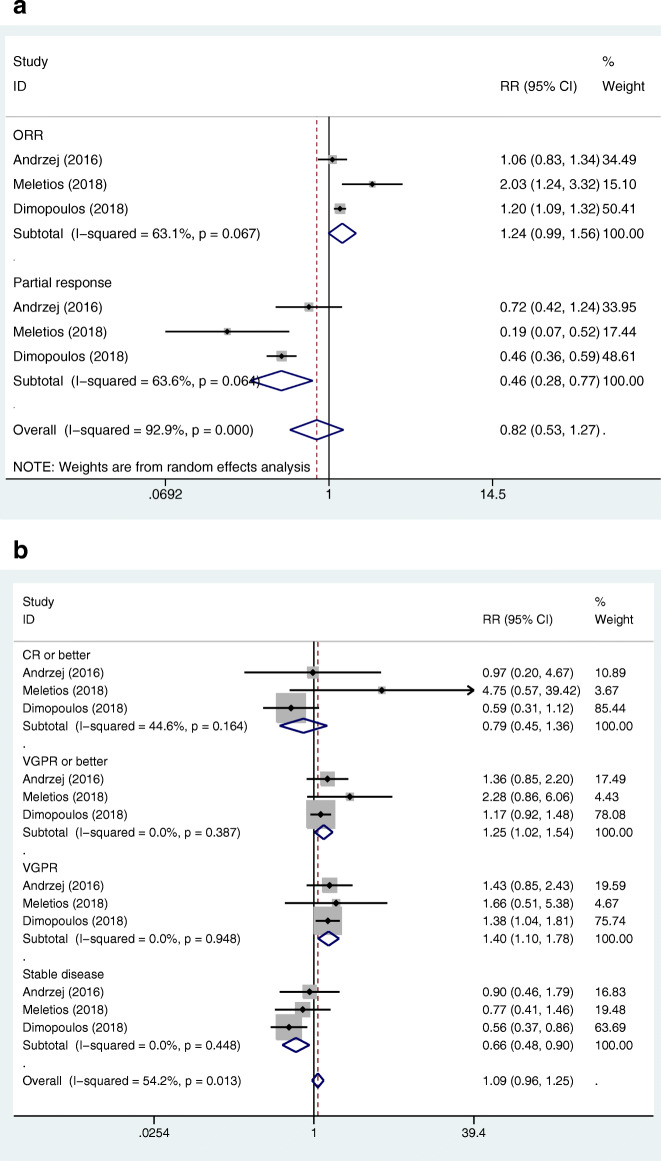
**a**. Forest plots of the pooled RRs for the ORR and partial response of the patients with MM in the MAbs targeting SLAMF7 group versus control group. The RR > 1 favours the MAb group. The size of the blocks or diamonds represents the weight of the random effects model in the meta-analysis. **b** Forest plots of the pooled RRs for the CR or better,VGPR or better, VGPR, and stable disease of the patients with MM in the MAbs targeting SLAMF7 group versus control group. The RR > 1 favours the MAb group. The size of the blocks or diamonds represents the weight of the fixed effects model in the meta-analysis

We used the same method as the above-mentioned, the pooled RRs for the ORR, CR or better, VGPR or better, VGPR, PR, and SD in the MAbs targeting CD38 group vs control group were 1.21 (95%CI 1.10–1.33), 1.78 (95%CI 1.61–1.98), 1.63 (95%CI 1.29–2.05), 1.39 (95%CI 1.02–1.89), 0.70 (95%CI 0.56–0.87), and 0.42 (95%CI 0.27–0.66), respectively (Fig. 4a, b). Further, we conducted subgroup analysis, according to the treatment status (primary or recurrent) and the classification of the antibodies, and found that there was no significant relationship between heterogeneity and the two factors. The pooled RRs for the ORR, CR or better, VGPR or better, VGPR, PR, and SD in the MAbs targeting SLAMF7 group vs control group were 1.24 (95%CI 0.99–1.56), 0.79 (95%CI 0.45–1.36), 1.25 (95%CI 1.02–1.54), 1.40 (95%CI 1.10–1.78), 0.46 (95%CI 0.28–0.77), and 0.66 (95%CI 0.48–0.90), respectively (Fig. 5a, b). The pooled RR for the ORR in the MAbs targeting PD-1/PD-L1 group vs control group was 0.97 (95%CI 0.82–1.13). The RRs for the ORR, CR or better, VGPR or better, VGPR, PR, and SD in the MAbs targeting CD38 group vs SLAMF7 group were 0.976 (95%CI 0.763–1.248), 2.253 (95%CI 1.284–3.955), 1.304 (95%CI 0.956–1.778), 0.993 (95%CI 0.671–1.468), 1.522 (95%CI 0.876–2.642), and 0.636 (95%CI 0.368–1.099) by indirect comparison, respectively. The RRs for the ORR in the MAbs targeting CD38 group vs PD-1/PD-L1 group and in the SLAMF7 group vs PD-1/PD-L1 group were 1.247 (95%CI 1.035–1.503) and 1.278 (95%CI 0.968–1.688), respectively. As for the treatment response, the MAbs targeting CD38 group was better than the MAbs targeting SLAMF7 group in terms of ‘CR or better’. The MAbs targeting PD-1/PD-L1 group had a worse treatment response compared to the MAbs targeting CD38 group and SLAMF7 group.

### Grade 3 or higher hematological and non-hematological adverse events

**Fig. 6:**
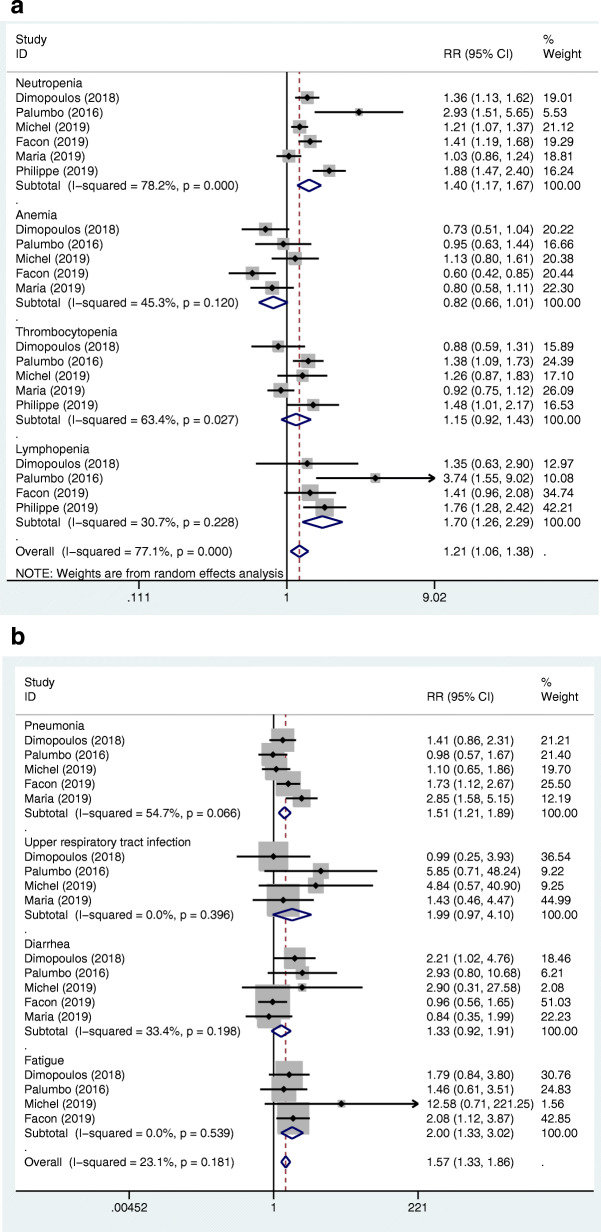
**a**. Forest plots of the pooled RRs for the grade 3 or higher hematological adverse events (including neutropenia, anemia, thrombocytopenia, and lymphopenia) of the patients with MM in the MAbs targeting CD38 group versus control group. The RR < 1 favours the MAb group. The size of the blocks or diamonds represents the weight of the random effects model in the meta-analysis. **b** Forest plots of the pooled RRs for the grade 3 or higher non-hematological adverse events (including pneumonia, upper respiratory tract infection, diarrhea, and fatigue) of the patients with MM in the MAbs targeting CD38 group versus control group. The RR < 1 favours the MAb group. The size of the blocks or diamonds represents the weight of the fixed effects model in the meta-analysis

**Fig. 7:**
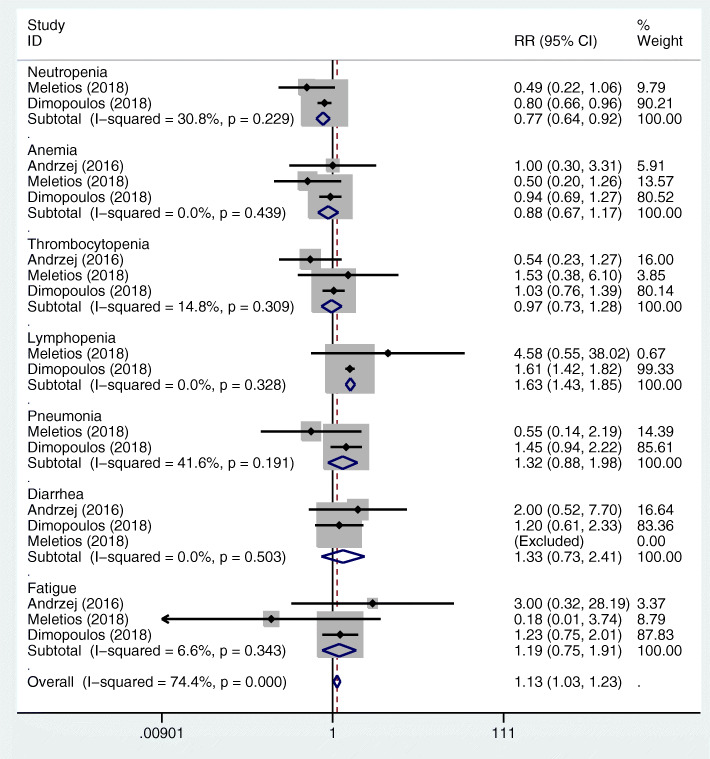
Forest plots of the pooled RRs for grade 3 or higher hematological (including neutropenia, anemia, thrombocytopenia, and lymphopenia) and non-hematological adverse events (including pneumonia, diarrhea, and fatigue) of the patients with MM in the MAbs targeting SLAMF7 group versus control group. The RR < 1 favours the MAb group. The size of the blocks or diamonds represents the weight of the fixed effects model in the meta-analysis

**Fig. 8:**
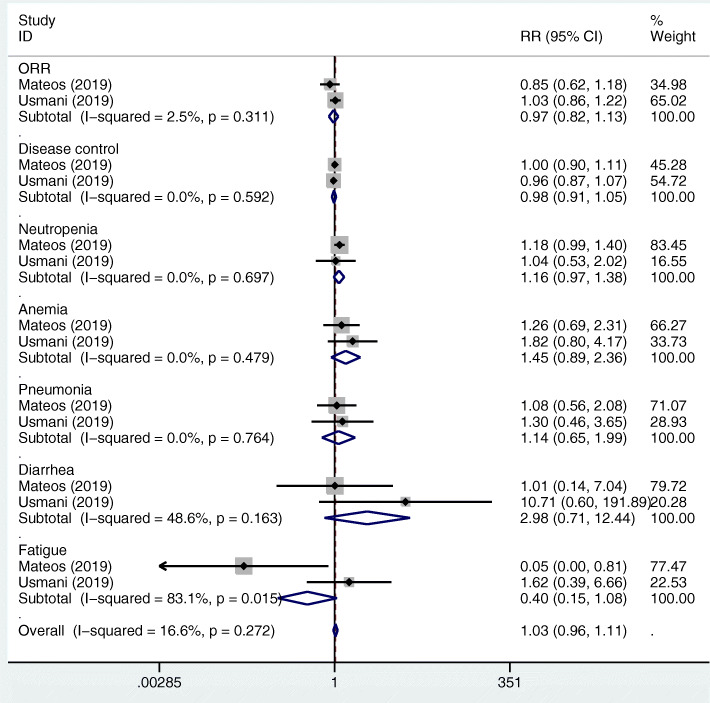
Forest plots of the pooled RRs for the ORR, disease control, and grade 3 or higher hematological (including neutropenia, and anemia) and non-hematological (including pneumonia, diarrhea, and fatigue) adverse events of the patients with MM in the MAbs targeting PD-1/PD-L1 group versus control group. The RRs for the ORR and disease control > 1 favour the MAb group. The RRs for the incidence of adverse events < 1 favour the MAb group. The size of the blocks or diamonds represents the weight of the fixed effects model in the meta-analysis

The pooled RRs for neutropenia, anemia, thrombocytopenia, lymphopenia, pneumonia, diarrhea, and fatigue in the MAbs targeting CD38 group vs control group were 1.40 (95%CI 1.17–1.67), 0.82 (95%CI 0.66–1.01), 1.15 (95%CI 0.92–1.43), 1.70 (95%CI 1.26–2.29), 1.51 (95%CI 1.21–1.89), 1.33 (95%CI 0.92–1.91), and 2.00 (95%CI 1.33–3.02) (Fig. 6a, b). The pooled RRs for neutropenia, anemia, thrombocytopenia, lymphopenia, pneumonia, diarrhea, and fatigue in the MAbs targeting SLAMF7 group vs control group were 0.77 (95%CI 0.64–0.92), 0.88 (95%CI 0.67–1.17), 0.97 (95%CI 0.73–1.28), 1.63 (95%CI 1.43–1.85), 1.32 (95%CI 0.88–1.98), 1.33 (95%CI 0.73–2.41), and 1.19 (95%CI 0.75–1.91), respectively (Fig. 7). The pooled RRs for neutropenia, anemia, pneumonia, diarrhea, and fatigue in the MAbs targeting PD-1/PD-L1 group vs control group were 1.16 (95%CI 0.97–1.38), 1.45 (95%CI 0.89–2.36), 1.14 (95%CI 0.65–1.99), 2.98 (95%CI 0.71–12.44), and 0.40 (95%CI 0.15–1.08), respectively (Fig. 8). The RRs for neutropenia, anemia, thrombocytopenia, lymphopenia, pneumonia, diarrhea, and fatigue in the MAbs targeting CD38 group vs SLAMF7 group were 1.818 (95%CI 1.41–2.344), 0.932 (95%CI 0.656–1.323), 1.186 (95%CI 0.83–1.694), 1.043 (95%CI 0.753–1.444), 1.144 (95% CI0.72–1.817), 1.00 (95%CI 0.497–2.014), and 1.681 (95%CI 0.903–3.13), respectively. The RRs for neutropenia, anemia, pneumonia, diarrhea, and fatigue in the MAbs targeting CD38 group vs PD-1/PD-L1 group by indirect comparison were 1.207 (95%CI 0.94–1.55), 0.566 (95%CI 0.332–0.963), 1.325 (95%CI 0.725–2.419), 0.446 (95%CI 0.102–1.956), and 5.00 (95%CI 1.717–14.56), respectively. The RRs for neutropenia, anemia, pneumonia, diarrhea, and fatigue in the MAbs targeting SLAMF7 group vs PD-1/PD-L1 group were 0.664 (95%CI 0.515–0.855), 0.607 (95%CI 0.346–1.064), 1.158 (95%CI 0.58–2.311), 0.446 (95%CI 0.095–2.105), and 2.975 (95%CI 0.998–8.867), respectively. As for the incidence of the adverse events, the MAbs targeting CD38 group exhibited a lower risk of anemia but a higher risk of fatigue than the MAbs targeting PD-1/PD-L1 group. The MAbs targeting SLAMF7 group had a lower risk of neutropenia than the MAbs targeting PD-1/ PD-L1 and CD38 groups.

The indirect-comparison results of efficacy and safety among the three groups were summarized in Table 3.

**Table 3:** The indirect-comparison results of efficacy and safety of MAbs targeting CD38, SLAMF7 and PD-1/PD-L1 groups

Outcomes	Antibodies targeting CD38 vs SLAMF7	Antibodies targeting CD38 vs PD-1/PD-L1	Antibodies targeting SLAMF7 vs PD-1/PD-L1
PFR - HR(95CI)	0.662 (0.543,0.806)	0.317 (0.221,0.454)	0.479 (0.328,0.699)
OS - HR(95CI)	0.812 (0.584,1.127)	NA	NA
ORR - RR(95CI)	0.976 (0.763,1.248)	1.247 (1.035,1.503)	1.278 (0.968,1.688)
CR or better-RR(95CI)	2.253 (1.284,3.955)	NA	NA
VGPR or better-RR(95CI)	1.304 (0.956,1.778)	NA	NA
VGPR - RR(95CI)	0.993 (0.671,1.468)	NA	NA
PR- RR(95CI)	1.522 (0.876,2.642)	NA	NA
SD - RR(95CI)	0.636 (0.368,1.099)	NA	NA
Neutropenia - RR(95CI)	1.818 (1.41,2.344)	1.207 (0.94,1.55)	0.664 (0.515,0.855)
Anemia-RR(95CI)	0.932 (0.656,1.323)	0.566 (0.332,0.963)	0.607 (0.346,1.064)
Thrombocytopenia -RR(95CI)	1.186 (0.83,1.694)	NA	NA
Lymphopenia -RR(95CI)	1.043 (0.753,1.444)	NA	NA
Pneumonia-RR(95CI)	1.144 (0.72,1.817)	1.325 (0.725,2.419)	1.158 (0.58,2.311)
Diarrhea-RR(95CI)	1.00 (0.497,2.014)	0.446 (0.102,1.956)	0.446 (0.095,2.105)
Fatigue-RR(95CI)	1.681 (0.903,3.13)	5.00 (1.717,14.56)	2.975 (0.998,8.867)

*Abbreviations*: *PFS* progression-free survival, *OS* overall survival, *ORR* overall response rate, *CR* complete response, *VGPR* very good partial response, *PR* partial response, *SD* stable disease, *HR* hazard ratio, *RR* relative risk, *NA* not available

## Discussion

MM is an incurable hematological malignancy with various clinical manifestations and outcomes [29]. With the use of new agents such as bortezomib and lenalidomide, the clinical outcomes of MM patients have dramatically improved. Unfortunately, most patients with MM eventually relapsed even after CR, which brings great challenge to the treatment of the disease [12, 30]. The application of MAbs brings new hope for the treatment of MM. CD38 was highly expressed in MM cells but less expressed on normal cells; this makes it a promising target for immunotherapy [29]. The anti-CD38 MAbs, including daratumumab, MOR202, and isatuximab, were well tolerated, and achieved PR or better in about 30% of patients with MM as a single agent, and are promising partners in combination therapy [4, 31]. The SLAMF7 was expressed at a high level on MM and natural killer (NK) cells [8]. The MAbs targeting SLAMF7 such as elotuzumab are able to activate NK cells and enhance antibody-dependent cell-mediated cytotoxicity, making SLAMF7 an attractive target in tumour immunotherapy [4]. The clinical outcomes of patients with MM have been improved by the application of elotuzumab in combination with other agents [7]. In MM, immune disorders have become a significant part of novel therapeutic strategies [10]. As an immune checkpoint receptor, PD-1 regulates the activity of T-cells by interacting with PD-L1 and PD-L2 [32]. Specifically speaking, PD-1 binding with PD-L1 on the surface of MM cells inhibits T cell-proliferation and contributes to the immune escape of cancer cells [10]. In the treatment of MM, it seems to be an appropriate approach based on combination therapy on the condition that monotherapy with PD-1/PD-L1 MAbs had yielded unsatisfactory outcomes [33, 34]; the MAbs targeting PD-1/PD-L1 include pembrolizumab, durvalumab, and nivolumab. This meta- analysis compared the effect and safety of the MAbs targeting CD38, SLAMF7, and PD-1/PD-L1 in combination with bortezomib/immunomodulators plus dexamethasone/prednisone in the treatment of MM. As for survival outcomes, the MAbs targeting CD38 group resulted in longer PFS than the MAbs targeting SLAMF7 and PD-1/PD-L1 groups; the MAbs targeting SLAMF7 group produced a longer PFS than the MAbs targeting PD-1/PD-L1 group. Based on these results, we can conclude that the MAbs targeting CD38 group had the best PFS followed by those targeting SLAMF7 group; the MAbs targeting PD-1/PD-L1 group was the worst by indirect comparison. As for the treatment responses, the MAbs targeting CD38 group elicited better treatment responses than the MAbs targeting SLAMF7 group in terms of ‘CR or better’, with no significant difference between the two with respect to ORR, VGPR or better, VGPR, PR, and SD. As for the incidence of grade 3 or higher hematological and non-hematological adverse events, the MAbs targeting CD38 group was associated with a higher incidence of neutropenia and a similar incidence of anemia, thrombocytopenia, lymphopenia, pneumonia, and diarrhea compared with the MAbs targeting SLAMF7 group. The MAbs targeting PD-1/PD-L1 group had a higher or similar incidence of adverse events (except fatigue) compared with the MAbs targeting CD38 group or SLAMF7 group.

To our knowledge, this is the first meta-analysis comparing the effect and safety of different MAbs in combination with other agents in the treatment of patients with MM. However, this meta-analysis has several limitations. First, the number of included studies testing the MAbs targeting SLAMF7 and PD-1/PD-L1 was insufficient, and heterogeneity existed among studies in the pooled RRs for the treatment responses of the MAbs groups vs corresponding controls. Second, the original data for each patient was not available and the abstracted data were from the published studies; meta-analysis based on individual patient data would be more valid for providing more reliable estimates. Third, we did not use sensitivity analysis to evaluate the impact of each study on the stability of the pooled effect by excluding individual studies one at a time because of the limited number of the included studies. Finally, for the same reason, we did not detect publication bias for the meta-analysis by using Egger’s and Begg’s tests.

## Conclusions

In general, the MAbs targeting CD38 in combination with bortezomib/immunomodulators plus dexamethasone/prednisone showed a significant therapeutic value in patients with MM. Although the treatment with the MAbs targeting SLAMF7 group was not as effective as that with the MAbs targeting CD38 group, it had a lower incidence of adverse events and may be more suitable for patients with poor drug tolerance. The therapeutic effect of the MAbs targeting PD-1/PD-L1 group was poor and the incidence of adverse events was not reduced or was even higher in comparison with the control or the other two groups, offering a limited therapeutic value to patients with MM. We found that different MAbs directing to different targets in combination with other agents had different effects; therefore, this study could provide a resource for clinicians when selecting an antibody to combine with other drugs for the treatment of MM.

## Supplementary Information

**Additional file 1: Table 1.** Characteristics of the patients at baseline. Table 2. Characteristics of the patients at baseline.

## Data Availability

Not applicable.

## Abbreviations

PFS: Progression-free survival
OS: Overall survival
ORR: Overall response rate
CR: Complete response
VGPR: Very good partial response
PR: Partial response
SD: Stable disease
HR: Hazard ratio
RR: Relative risk
MM: Multiple myeloma

## References

[1] Morandi F, Horenstein AL, Costa F, Giuliani N, Pistoia V, Malavasi F (2018). CD38: a target for immunotherapeutic approaches in multiple myeloma. Front Immunol.

[2] Chini EN, Chini C, Espindola NJ, de Oliveira GC, van Schooten W (2018). The pharmacology of CD38/NADase: an emerging target in Cancer and diseases of aging. Trends Pharmacol Sci.

[3] van de Donk N, Usmani SZ (2018). CD38 antibodies in multiple myeloma: mechanisms of action and modes of resistance. Front Immunol.

[4] Malaer JD, Mathew PA (2017). CS1 (SLAMF7, CD319) is an effective immunotherapeutic target for multiple myeloma. Am J Cancer Res.

[5] Malaer JD, Marrufo AM, Mathew PA (2019). 2B4 (CD244, SLAMF4) and CS1 (CD319, SLAMF7) in systemic lupus erythematosus and cancer. Clin Immunol.

[6] Chen J, Zhong MC, Guo H, Davidson D, Mishel S, Lu Y, Rhee I, Perez-Quintero LA, Zhang S, Cruz-Munoz ME, Wu N, Vinh DC, Sinha M, Calderon V, Lowell CA, Danska JS, Veillette A (2017). SLAMF7 is critical for phagocytosis of haematopoietic tumour cells via mac-1 integrin. NATURE.

[7] Pazina T, James AM, MacFarlane AT, Bezman NA, Henning KA, Bee C, Graziano RF, Robbins MD, Cohen AD, Campbell KS (2017). The anti-SLAMF7 antibody elotuzumab mediates NK cell activation through both CD16-dependent and -independent mechanisms. Oncoimmunology.

[8] Ishibashi M, Soeda S, Sasaki M, Handa H, Imai Y, Tanaka N, Tanosaki S, Ito S, Odajima T, Sugimori H, Asayama T, Sunakawa M, Kaito Y, Kinoshita R, Kuribayashi Y, Onodera A, Moriya K, Tanaka J, Tsukune Y, Komatsu N, Inokuchi K, Tamura H (2018). Clinical impact of serum soluble SLAMF7 in multiple myeloma. Oncotarget.

[9] Rosenblatt J, Avigan D (2017). Targeting the PD-1/PD-L1 axis in multiple myeloma: a dream or a reality?. BLOOD.

[10] Oliva S, Troia R, D'Agostino M, Boccadoro M, Gay F (2018). Promises and pitfalls in the use of PD-1/PD-L1 inhibitors in multiple myeloma. Front Immunol.

[11] Lesokhin AM, Bal S, Badros AZ (2019). Lessons learned from checkpoint blockade targeting PD-1 in multiple myeloma. Cancer Immunol Res.

[12] Zheng Y, Shen H, Xu L, Feng J, Tang H, Zhang N, Chen X, Gao G (2018). Monoclonal antibodies versus histone deacetylase inhibitors in combination with Bortezomib or Lenalidomide plus dexamethasone for the treatment of relapsed or refractory multiple myeloma: an indirect-comparison Meta-analysis of randomized controlled trials. J Immunol Res.

[13] Kodama S, Fujihara K, Horikawa C, Harada M, Ishiguro H, Kaneko M, Furukawa K, Matsubayashi Y, Matsunaga S, Shimano H, Tanaka S, Kato K, Sone H (2018). Network meta-analysis of the relative efficacy of bariatric surgeries for diabetes remission. Obes Rev.

[14] Mateos MV, Cavo M, Blade J, Dimopoulos MA, Suzuki K, Jakubowiak A, Knop S, Doyen C, Lucio P, Nagy Z, Pour L, Cook M, Grosicki S, Crepaldi A, Liberati AM, Campbell P, Shelekhova T, Yoon SS, Iosava G, Fujisaki T, Garg M, Krevvata M, Chen Y, Wang J, Kudva A, Ukropec J, Wroblewski S, Qi M, Kobos R, San-Miguel J (2020). Overall survival with daratumumab, bortezomib, melphalan, and prednisone in newly diagnosed multiple myeloma (ALCYONE): a randomised, open-label, phase 3 trial. LANCET.

[15] Spencer A, Lentzsch S, Weisel K, Avet-Loiseau H, Mark TM, Spicka I, Masszi T, Lauri B, Levin MD, Bosi A, Hungria V, Cavo M, Lee JJ, Nooka AK, Quach H, Lee C, Barreto W, Corradini P, Min CK, Scott EC, Chanan-Khan AA, Horvath N, Capra M, Beksac M, Ovilla R, Jo JC, Shin HJ, Sonneveld P, Soong D, Casneuf T, Chiu C, Amin H, Qi M, Thiyagarajah P, Sasser AK, Schecter JM, Mateos MV (2018). Daratumumab plus bortezomib and dexamethasone versus bortezomib and dexamethasone in relapsed or refractory multiple myeloma: updated analysis of CASTOR. Haematologica.

[16] Dimopoulos MA, San-Miguel J, Belch A, White D, Benboubker L, Cook G, Leiba M, Morton J, Ho PJ, Kim K, Takezako N, Moreau P, Kaufman JL, Sutherland HJ, Lalancette M, Magen H, Iida S, Kim JS, Prince HM, Cochrane T, Oriol A, Bahlis NJ, Chari A, O'Rourke L, Wu K, Schecter JM, Casneuf T, Chiu C, Soong D, Sasser AK, Khokhar NZ, Avet-Loiseau H, Usmani SZ (2018). Daratumumab plus lenalidomide and dexamethasone versus lenalidomide and dexamethasone in relapsed or refractory multiple myeloma: updated analysis of POLLUX. Haematologica.

[17] Dimopoulos MA, Lonial S, Betts KA, Chen C, Zichlin ML, Brun A, Signorovitch JE, Makenbaeva D, Mekan S, Sy O, Weisel K, Richardson PG (2018). Elotuzumab plus lenalidomide and dexamethasone in relapsed/refractory multiple myeloma: extended 4-year follow-up and analysis of relative progression-free survival from the randomized ELOQUENT-2 trial. Cancer Am Cancer Soc.

[18] Attal M, Richardson PG, Rajkumar SV, San-Miguel J, Beksac M, Spicka I, Leleu X, Schjesvold F, Moreau P, Dimopoulos MA, Huang JS, Minarik J, Cavo M, Prince HM, Mace S, Corzo KP, Campana F, Le-Guennec S, Dubin F, Anderson KC (2019). Isatuximab plus pomalidomide and low-dose dexamethasone versus pomalidomide and low-dose dexamethasone in patients with relapsed and refractory multiple myeloma (ICARIA-MM): a randomised, multicentre, open-label, phase 3 study. Lancet.

[19] Moreau P, Attal M, Hulin C, Arnulf B, Belhadj K, Benboubker L, Bene MC, Broijl A, Caillon H, Caillot D, Corre J, Delforge M, Dejoie T, Doyen C, Facon T, Sonntag C, Fontan J, Garderet L, Jie KS, Karlin L, Kuhnowski F, Lambert J, Leleu X, Lenain P, Macro M, Mathiot C, Orsini-Piocelle F, Perrot A, Stoppa AM, van de Donk NW, Wuilleme S, Zweegman S, Kolb B, Touzeau C, Roussel M, Tiab M, Marolleau JP, Meuleman N, Vekemans MC, Westerman M, Klein SK, Levin MD, Fermand JP, Escoffre-Barbe M, Eveillard JR, Garidi R, Ahmadi T, Zhuang S, Chiu C, Pei L, de Boer C, Smith E, Deraedt W, Kampfenkel T, Schecter J, Vermeulen J, Avet-Loiseau H, Sonneveld P (2019). Bortezomib, thalidomide, and dexamethasone with or without daratumumab before and after autologous stem-cell transplantation for newly diagnosed multiple myeloma (CASSIOPEIA): a randomised, open-label, phase 3 study. Lancet.

[20] Facon T, Kumar S, Plesner T, Orlowski RZ, Moreau P, Bahlis N, Basu S, Nahi H, Hulin C, Quach H, Goldschmidt H, O'Dwyer M, Perrot A, Venner CP, Weisel K, Mace JR, Raje N, Attal M, Tiab M, Macro M, Frenzel L, Leleu X, Ahmadi T, Chiu C, Wang J, Van Rampelbergh R, Uhlar CM, Kobos R, Qi M, Usmani SZ (2019). Daratumumab plus Lenalidomide and dexamethasone for untreated myeloma. N Engl J Med.

[21] Mateos MV, Dimopoulos MA, Cavo M, Suzuki K, Jakubowiak A, Knop S, Doyen C, Lucio P, Nagy Z, Kaplan P, Pour L, Cook M, Grosicki S, Crepaldi A, Liberati AM, Campbell P, Shelekhova T, Yoon SS, Iosava G, Fujisaki T, Garg M, Chiu C, Wang J, Carson R, Crist W, Deraedt W, Nguyen H, Qi M, San-Miguel J (2018). Daratumumab plus Bortezomib, Melphalan, and prednisone for untreated myeloma. N Engl J Med.

[22] Dimopoulos MA, Oriol A, Nahi H, San-Miguel J, Bahlis NJ, Usmani SZ, Rabin N, Orlowski RZ, Komarnicki M, Suzuki K, Plesner T, Yoon SS, Ben YD, Richardson PG, Goldschmidt H, Reece D, Lisby S, Khokhar NZ, O'Rourke L, Chiu C, Qin X, Guckert M, Ahmadi T, Moreau P (2016). Daratumumab, Lenalidomide, and dexamethasone for multiple myeloma. N Engl J Med.

[23] Palumbo A, Chanan-Khan A, Weisel K, Nooka AK, Masszi T, Beksac M, Spicka I, Hungria V, Munder M, Mateos MV, Mark TM, Qi M, Schecter J, Amin H, Qin X, Deraedt W, Ahmadi T, Spencer A, Sonneveld P (2016). Daratumumab, Bortezomib, and dexamethasone for multiple myeloma. N Engl J Med.

[24] Lonial S, Dimopoulos M, Palumbo A, White D, Grosicki S, Spicka I, Walter-Croneck A, Moreau P, Mateos MV, Magen H, Belch A, Reece D, Beksac M, Spencer A, Oakervee H, Orlowski RZ, Taniwaki M, Rollig C, Einsele H, Wu KL, Singhal A, San-Miguel J, Matsumoto M, Katz J, Bleickardt E, Poulart V, Anderson KC, Richardson P (2015). Elotuzumab therapy for relapsed or refractory multiple myeloma. N Engl J Med.

[25] Jakubowiak A, Offidani M, Pegourie B, De La Rubia J, Garderet L, Laribi K, Bosi A, Marasca R, Laubach J, Mohrbacher A, Carella AM, Singhal AK, Tsao LC, Lynch M, Bleickardt E, Jou YM, Robbins M, Palumbo A (2016). Randomized phase 2 study: elotuzumab plus bortezomib/dexamethasone vs bortezomib/dexamethasone for relapsed/refractory MM. BLOOD.

[26] Dimopoulos MA, Dytfeld D, Grosicki S, Moreau P, Takezako N, Hori M, Leleu X, LeBlanc R, Suzuki K, Raab MS, Richardson PG, Popa MM, Jou YM, Shelat SG, Robbins M, Rafferty B, San-Miguel J (2018). Elotuzumab plus Pomalidomide and dexamethasone for multiple myeloma. N Engl J Med.

[27] Usmani SZ, Schjesvold F, Oriol A, Karlin L, Cavo M, Rifkin RM, Yimer HA, LeBlanc R, Takezako N, McCroskey RD, Lim A, Suzuki K, Kosugi H, Grigoriadis G, Avivi I, Facon T, Jagannath S, Lonial S, Ghori RU, Farooqui M, Marinello P, San-Miguel J (2019). Pembrolizumab plus lenalidomide and dexamethasone for patients with treatment-naive multiple myeloma (KEYNOTE-185): a randomised, open-label, phase 3 trial. Lancet Haematol.

[28] Mateos MV, Blacklock H, Schjesvold F, Oriol A, Simpson D, George A, Goldschmidt H, Larocca A, Chanan-Khan A, Sherbenou D, Avivi I, Benyamini N, Iida S, Matsumoto M, Suzuki K, Ribrag V, Usmani SZ, Jagannath S, Ocio EM, Rodriguez-Otero P, San MJ, Kher U, Farooqui M, Liao J, Marinello P, Lonial S (2019). Pembrolizumab plus pomalidomide and dexamethasone for patients with relapsed or refractory multiple myeloma (KEYNOTE-183): a randomised, open-label, phase 3 trial. Lancet Haematol.

[29] Bonello F, D'Agostino M, Moscvin M, Cerrato C, Boccadoro M, Gay F (2018). CD38 as an immunotherapeutic target in multiple myeloma. Expert Opin Biol Ther.

[30] Yang WC, Lin SF (2015). Mechanisms of drug resistance in relapse and refractory multiple myeloma. Biomed Res Int.

[31] van de Donk N, Richardson PG, Malavasi F (2018). CD38 antibodies in multiple myeloma: back to the future. BLOOD.

[32] Korkmaz S, Erdem S, Akay E, Tasdemir EA, Karaman H, Keklik M (2019). Do PD-1 and PD-L2 expressions have prognostic impact in hematologic malignancies?. Turk J Med Sci.

[33] Jelinek T, Paiva B, Hajek R (2018). Update on PD-1/PD-L1 inhibitors in multiple myeloma. Front Immunol.

[34] Jelinek T, Mihalyova J, Kascak M, Duras J, Hajek R (2017). PD-1/PD-L1 inhibitors in haematological malignancies: update 2017. Immunology.

