# Supplementary material for: Comparison of monoclonal antibodies targeting CD38, SLAMF7 and PD-1/PD-L1 in combination with Bortezomib/Immunomodulators plus dexamethasone/prednisone for the treatment of multiple myeloma: an indirect-comparison Meta-analysis of randomised controlled trials
Source: BMC Cancer. 2021 Sep 6;21:994. doi: 10.1186/s12885-021-08588-9 (PMC8419924; doi:10.1186/s12885-021-08588-9)
Supplement: Supplementary file 1 — Additional file 1: Table 1. Characteristics of the patients at baseline. Table 2. Characteristics of the patients at baseline. [file 12885_2021_8588_MOESM1_ESM.docx]

Table 1 Characteristics of the patients at baseline.

| Study | POLLUX  T C | CASTOR  T C | NCT02252172  T C | ALCYONE  T C | ICARIA-MM  T C | CASSIOPEIA  T C |
| --- | --- | --- | --- | --- | --- | --- |
| Number of patients | 286 283 | 251 247 | 368 369 | 350 356 | 154 153 | 543 524 |
| Median age(year) | 65 65 | 64 64 | 73 74 | 71 71 | 68 66 | 59 58 |
| ECOG^‡^performance status -no. (%) |  |  |  |  |  |  |
| 0 | 139(48.6) 150(53.0) | NA NA | 127(34.5) 123(33.3) | 78(22.3) 99(27.8) | NA NA | 265(49) 257(47) |
| 1 | NA NA | NA NA | 178(48.4) 187 (50.7) | 182(52.0) 173(48.6) | NA NA | 225(41) 230(42) |
| 2 | NA NA | NA NA | 63 (17.1) 59 (16.0) | 90 (25.7) 84 (23.6) | NA NA | 53(10) 55(10) |
| ISS^§^ disease stage -no. (%) |  |  |  |  |  |  |
| 1 | 137(47.9) 140(49.5) | 98(39.0) 96(38.9) | 98 (26.6) 103 (27.9) | 69 (19.7) 67 (18.8) | 64 (42) 51 (33) | 204(38) 228(42) |
| 2 | 93 (32.5) 86 (30.4) | 94(37.5) 100(40.5) | 163(44.3) 156 (42.3) | 139(39.7) 160 (44.9) | 53(34) 56 (37) | 255(47) 233(43) |
| 3 | 56 (19.6) 57 (20.1) | 59(23.5) 51 (20.6) | 107(29.1) 110 (29.8) | 142(40.6) 129 (36.2) | 34(22) 43 (28) | 84(15) 81(15) |
| Cytogenetic profile -no. (%) |  |  |  |  |  |  |
| Standard risk | T:193/228(84.6)  C: 176/211(83.4) | T:140/181(77.3) C:137/174 (78.7) | T:271/319(85.0) C:279/323 (86.4) | T:261/314(83.1) C:257/302 (85.1) | 103(67) 78(51) | T:460/542(85) C:454/540 (84) |
| High risk | T:35/228(15.4) C:35/211 (16.6) | T:41/181(22.7) C:37/174 (21.3) | T:48/319(15.0)  C:44/323 (13.6) | T:53/314(16.9) C:45/302 (14.9) | 24(16) 36 (24) | T:82/542(15) C:86/540 (16) |
| Median time since initial diagnosis | 3.5yr 4.0yr | 3.87yr 3.72yr | 0.95M 0.89 M | 0.8M 0.8M | 4.46yr 4.09yr | 0.92M 0.92M |
| Median no. of previous lines of therapy | 1 1 | 2 2 | 0 0 | 0 0 | 3 3 | 0 0 |

‡ Eastern Cooperative Oncology Group (ECOG) performance status is scored on a scale from 0 to 5, with 0 indicating no symptoms and higher scores indicating increasing disability.

§ The International Staging System (ISS) disease stage is derived on the basis of the combination of serum β2-microglobulin and albumin levels. Higher stages indicate more advanced disease.

Abbreviation：NA, not available T, trail C, control M, month

Table 2 Characteristics of the patients at baseline.

| Study | KEYNOTE-183  T C | KEYNOTE-185  T C | NCT01478048  T C | ELOQUENT-3  T C | ELOQUENT-2  T C |
| --- | --- | --- | --- | --- | --- |
| Number of patients | 125 124 | 151 150 | 77 75 | 60 57 | 321 325 |
| Median age(year) | 65 67 | 74 74 | 65 65 | 69 66 | 67 66 |
| ECOG^‡^ performance status -no. (%) |  |  |  |  |  |
| 0 | 60 (48) 60 (48) | 51 (34) 55 (37) | 38 (49) 46 (61) | NA NA | NA NA |
| 1 | 65 (52) 64 (52) | 100 (66) 92(61) | 35 (46) 23 (31) | NA NA | NA NA |
| 2 | 0 0 | 0 1 (1) | 2 (3) 6 (8) | NA NA | NA NA |
| ISS^§^ disease stage -no. (%) |  |  |  |  |  |
| 1 | 45 (36) 45 (36) | 38 (25) 51 (34) | 26 (34) 19 (25) | (1 or 2)  53 (88) 50 (88) | 141(44) 138 (42) |
| 2 | 46 (37) 39 (31) | 68 (45) 66 (44) | 23 (30) 20 (27) |  | 102(32) 105(32) |
| 3 | 33 (26) 33 (27) | 44 (29) 31 (21) | 11 (14) 16 (21) | 7 (12) 7 (12) | 66 (21) 68 (21) |
| Cytogenetic profile -no. (%) |  |  |  |  |  |
| Standard risk | 52 (42) 71 (57) | 93 (62) 89 (59) | NA NA | NA NA | NA NA |
| High risk | 28 (22) 17 (14) | 24 (16) 10 (7) | NA NA | NA NA | NA NA |
| Median time since initial diagnosis | NA NA | NA NA | 45M 44M | 4.8yr 4.4yr | NA NA |
| Median no. of previous lines of therapy | 3 3 | 0 0 | NA NA | 3 3 | 2 2 |

‡ Eastern Cooperative Oncology Group (ECOG) performance status is scored on a scale from 0 to 5, with 0 indicating no symptoms and higher scores indicating increasing disability.

§ The International Staging System (ISS) disease stage is derived on the basis of the combination of serum β2-microglobulin and albumin levels. Higher stages indicate more advanced disease.

Abbreviation：NA, not available T, trail C, control M, month
